# Heterotrimeric G‐protein subunit G*α*_i2_ contributes to agonist‐sensitive apoptosis and degranulation in murine platelets

**DOI:** 10.14814/phy2.13841

**Published:** 2018-09-05

**Authors:** Hang Cao, Syed M. Qadri, Elisabeth Lang, Lisann Pelzl, Anja T. Umbach, Veronika Leiss, Lutz Birnbaumer, Bernd Nürnberg, Burkert Pieske, Jakob Voelkl, Meinrad Gawaz, Rosi Bissinger, Florian Lang

**Affiliations:** ^1^ Department of Vegetative & Clinical Physiology Eberhard‐Karls University Tübingen Germany; ^2^ Department of Pathology and Molecular Medicine McMaster University Hamilton Ontario Canada; ^3^ Centre for Innovation Canadian Blood Services Hamilton Ontario Canada; ^4^ Department of Molecular Medicine II Heinrich Heine University Düsseldorf Germany; ^5^ Department of Pharmacology and Experimental Therapy Interfaculty Center of Pharmacology and Drug Research (ICePhA) Eberhard‐Karls University Tübingen Germany; ^6^ Neurobiology Laboratory National Institute of Environmental Health Sciences National Institutes of Health Durham North Carolina; ^7^ Institute of Biomedical Research (BIOMED) Catholic University of Argentina Buenos Aires Argentina; ^8^ Department of Internal Medicine and Cardiology Charité‐Universitätsmedizin Berlin, and German Heart Institute Berlin Germany; ^9^ Berlin Institute of Health (BIH) Berlin Germany; ^10^ DZHK (German Centre for Cardiovascular Research), partner site Berlin Germany; ^11^ Department of Internal Medicine III Eberhard‐Karls University Tübingen Germany

**Keywords:** Apoptosis, degranulation, G‐protein, G*α*_i2_, platelets

## Abstract

G*α*_i2_, a heterotrimeric G‐protein subunit, regulates various cell functions including ion channel activity, cell differentiation, proliferation and apoptosis. Platelet‐expressed G*α*_i2_ is decisive for the extent of tissue injury following ischemia/reperfusion. However, it is not known whether G*α*_i2_ plays a role in the regulation of platelet apoptosis, which is characterized by caspase activation, cell shrinkage and cell membrane scrambling with phosphatidylserine (PS) translocation to the platelet surface. Stimulators of platelet apoptosis include thrombin and collagen‐related peptide (CoRP), which are further known to enhance degranulation and activation of *α* _II__b_ *β*3‐integrin and caspases. Using FACS analysis, we examined the impact of agonist treatment on activation and apoptosis in platelets drawn from mice lacking G*α*_i2_ and their wild‐type (WT) littermates. As a result, treatment with either thrombin (0.01 U/mL) or CoRP (2 *μ*g/mL or 5 *μ*g/mL) significantly upregulated PS‐exposure and significantly decreased forward scatter, reflecting cell size, in both genotypes. Exposure to CoRP triggered a significant increase in active caspase 3, ceramide formation, surface P‐selectin, and *α* _II__b_ *β*3‐integrin activation. These molecular alterations were significantly less pronounced in G*α*_i2_‐deficient platelets as compared to WT platelets. In conclusion, our data highlight a previously unreported role of G*α*_i2_ signaling in governing platelet activation and apoptosis.

## Introduction

Heterotrimeric G proteins are coupled to plasma membrane receptors and participate in the regulation of a wide range of cellular functions such as migration, differentiation, proliferation, ion channel activity, and apoptosis (Hilger et al. 2018; Squires et al. 2018). In platelets, fundamental elements of the hemostasis machinery, a wide array of cell surface receptors coupled to G proteins mediate their activation (Offermanns 2006). The P2Y_12_ receptor, an important therapeutic target contributing to purinergic stimulation of platelets, is coupled to heterotrimeric G*α*_i2_ protein, the predominant G*α* isoform (Gachet 2012). More recently, G*α*_i2_ protein was reported to play a thrombo‐inflammatory role in orchestrating thrombus stabilization during hemostasis and mediating tissue damage during experimental cerebral and myocardial ischemia‐reperfusion injury (Devanathan et al. 2015).

Platelets are activated by a wide range of agonists such as subendothelial collagen, thrombin, ADP secreted from stimulated platelets, and by collagen‐related peptide (CoRP) (Lang et al. 2013). In the presence of stimulatory signals, platelets degranulate, undergo phospholipid scrambling on their cell membrane, and aggregate to form thrombi that results in vascular blockage (Varga‐Szabo et al. 2009; Munzer et al. 2014). Cell membrane scrambling with phosphatidylserine (PS) exposure fosters the assembly of tenase and prothrombinase complexes, and subsequently promoting factor Xa and thrombin generation (Lebois and Josefsson 2016). In platelets, unconstrained apoptosis has been implicated in thrombocytopenia, bleeding disorders, microparticle shedding as well as affecting their quality during storage (Lebois and Josefsson 2016; Quach et al. 2018). PS exposure in apoptotic platelets, triggered by exogenous agents or physiological changes, leads to their clearance from the circulation (Lebois and Josefsson 2016; Quach et al. 2018). G*α*_i2_ has previously been shown to participate in the apoptosis machinery of different cell types (Lopez‐Aranda et al. 2008; Bissinger et al. 2016). Despite the recent evidence linking G*α*_i2_ protein to the execution of thrombosis, its role in platelet survival has hitherto remained elusive. In the present study, using thrombin and collagen‐related peptide (CoRP), an agonist mimicking the effect of contact with collagen and a powerful stimulator of platelet apoptosis (Munzer et al. 2018), we investigated the influence of G*α*_i2_ on platelet survival and degranulation ex vivo.

## Materials and Methods

### Mice

Experiments were performed in G*α*_i2_‐deficient mice (*Gα*_*i2*_^*−/−*^) and their wild‐type (WT) littermates of 10–12 weeks of age. The mice were generated and initially characterized on a SV129 background (Rudolph et al. 1995). Mice were backcrossed on a C57BL/6 background and kept under specified pathogen‐free (SPF) environment in individually ventilated cages (IVC) to prolong life expectancy (Wiege et al. 2013; Devanathan et al. 2015). All animal experiments were conducted according to the German law for the care and use of laboratory animals and were approved by local government authorities (Regierungspräsidium Tübingen according to §4 of 19/12/2011).

### Preparation of mouse platelets

Platelets were obtained from 10‐ to 12‐week‐old mice of either sex which were treated according to the protocol approved by government authorities. Eight hundred *μ*l blood was obtained into tubes containing 200 *μ*l acid‐citrate‐dextrose buffer. Platelet rich plasma (PRP) was obtained by centrifugation at 260 g for 5 min. PRP was then centrifuged at 640 g for 5 min to pellet the platelets. Where necessary, apyrase (0.02 U/mL; Sigma‐Aldrich) and prostaglandin I2 (0.5 *μ*mol/L; Calbiochem) were added to the PRP to prevent activation of platelets during isolation. After two washing steps, the pellet of washed platelets was resuspended in modified Tyrode‐HEPES buffer (pH 7.4, supplemented with 1 mmol/L CaCl_2_). Where indicated, thrombin (0.01 U/mL, Roche, Basel, Switzerland) or collagen‐related peptide (CoRP, 2 *μ*g/mL or 5 *μ*g/mL, kindly provided by R. Farndale, University of Cambridge, Cambridge, UK) were added (Liu et al. 2016).

### Cytosolic calcium

For the measurement of the cytosolic Ca^2+^ concentration, the platelet preparation was washed once in Tyrode buffer (pH 7.4), stained with 3 *μ*mol/L Fluo‐3AM (Biotium, USA) in the same buffer and incubated at 37°C for 30 min. Following the indicated experimental treatment, relative fluorescence was measured utilizing a BD FACS Calibur (BD Biosciences, Heidelberg, Germany) (Towhid et al. 2011; Liu et al. 2016).

### P‐selectin and activated integrin abundance

Fluorophore‐labeled antibodies were utilized for the detection of P‐selectin expression (Wug.E9‐FITC, Emfret Analytics, Eibelstadt, Germany) and the active form of *α*_IIb_ *β*3 integrin (JON/A‐PE, Emfret Analytics, Eibelstadt, Germany). Washed mouse platelets (1x10^6^) were suspended in modified Tyrode buffer (pH 7.4) containing 1 mmol/L CaCl_2_ and antibodies (1:10 dilution) and subsequently subjected to the respective treatments and for the indicated time periods at room temperature (RT). The reaction was stopped by addition of PBS and the samples were immediately analyzed by FACS analysis (Liu et al. 2015).

### Phosphatidylserine exposure and forward scatter

Phosphatidylserine exposure was determined in platelets with and without a 10 min thrombin or CoRP treatment (Liu et al. 2016). To this end, the platelet preparation was centrifuged at 660 g for 5 min followed by washing once with Tyrode buffer (pH 7.4) with 1 mmol/L CaCl_2_, staining with 1:20 dilution of Annexin‐V FITC (ImmunoTools, Germany) in Tyrode buffer (pH 7.4) in the presence of 2 mmol/L CaCl_2_ and incubation at 37°C for 30 min. Annexin‐V binding reflecting surface exposure of phosphatidylserine was evaluated by FACS analysis. In parallel, the forward scatter (FSC) of the platelets was determined by FACS analysis, as a measure of platelet size (Liu et al. 2016).

### Caspase‐3 activity

Caspase 3 activity was determined utilizing a CaspGlow Fluorescein Active Caspase‐3 Staining kit from BioVision (CA, USA) according to the manufacturer's instruction. Fluorescence intensity was measured at an excitation wavelength of 488 nm and an emission wavelength of 530 by FACS analysis.

### Ceramide abundance

Ceramide abundance at platelet cell surface was measured by a monoclonal antibody‐assay. For this purpose, platelets were stained at 37°C for 1 h at a concentration of 1 *μ*g/mL anti‐ceramide antibody (clone MID 15B4, Enzo Life Science GmbH, Lörrach, Germany) in Tyrode buffer at a dilution of 1:10. Platelets were washed once with Tyrode buffer. Subsequently, platelets were stained for 30 min with polyclonal fluorescein isothiocyanate (FITC) conjugated goat anti‐mouse IgG and IgM specific antibody (BD Pharmingen, Hamburg, Germany) at a concentration of 1:50 in Tyrode buffer. Washing of the platelets was performed in order to remove unbound secondary antibody. Finally, the samples were analyzed by flow cytometry at an excitation wavelength of 488 nm and an emission wavelength of 530 nm, as has previously been shown (Gatidis et al. 2010).

### Statistical analysis

Data are shown as means ± SD; n represents the number of independent experiments. All data were tested for significance using ANOVA. *P* < 0.05 was considered statistically significant.

## Results

This study addressed the impact of G*α*_i2_ on activation and apoptosis of murine platelets. For this purpose, experiments were performed in mice lacking G*α*_i2_ and corresponding wild type mice. The platelets were analyzed with or without prior exposure to thrombin (0.01 U/mL) or collagen‐related peptide (CoRP, 2 *μ*g/mL and 5 *μ*g/mL). Individual values (mean ± SD) of female and male WT and G*α*_i2_‐deficient mice of annexin‐V‐binding, platelet volume, caspase‐3‐positive cells, ceramide abundance, P‐selectin abundance and activated integrin *α*_IIb_ *β*3 are displayed in Table 1A–D. No significant differences were observed between male and female WT or G*α*_i2_‐deficient mice.

**Table 1:** Analyzed parameters in WT and G*α*_i2_‐deficient mice

A: Annexin‐V‐binding and forward scatter in WT female and male mice						
Parameter	WT (female)			WT (male)		
	Resting	Thrombin	CoRP	Resting	Thrombin	CoRP
Annexin‐V‐binding [%]	0.89 ± 0.02	17.07 ± 2.50	19.22 ± 0.87	1.06 ± 0.39	18.22 ± 2.34	18.60 ± 2.75
Forward Scatter [Geomean; arb. units]	14.95 ± 1.00	10.43 ± 0.95	8.15 ± 0.18	15.09 ± 0.46	11.29 ± 1.03	9.42 ± 1.11

B: Annexin‐V‐binding and forward scatter in G*α*_i2_‐deficient female and G*α*_i2_‐deficient male mice						
Parameter	G*α*_i2_‐deficient (female)			G*α*_i2_‐deficient (male)		
	Resting	Thrombin	CoRP	Resting	Thrombin	CoRP
Annexin‐V‐binding [%]	1.13 ± 0.17	13.65 ± 0.66	8.75 ± 2.84	1.26 ± 0.53	14.11 ± 0.99	10.32 ± 0.98
Forward Scatter [Geomean; arb. units]	15.94 ± 0.53	12.04 ± 0.87	11.10 ± 0.86	16.68 ± 0.58	13.30 ± 0.55	11.97 ± 0.46

C: P‐selectin, activated integrin *α*_IIb_ *β*3, caspase‐3‐positive cells and ceramide abundance in WT female and WT male mice				
Parameter	WT (female)		WT (male)	
	Resting	CoRP	Resting	CoRP
P‐selectin [MFI]	5.29 ± 0.43	28.01 ± 5.63	5.74 ± 0.39	31.86 ± 3.10
Activated integrin *α*_IIb_ *β*3 [MFI]	5.59 ± 0.27	96.61 ± 15.64	5.29 ± 0.50	88.90 ± 0.83
Caspase‐3‐positive cells [%]	4.65 ± 0.60	21.91 ± 1.65	4.28 ± 0.23	22.69 ± 2.16
Ceramide abundance [MFI]	19.38 ± 2.28	25.48 ± 0.28	21.09 ± 0.08	24.91 ± 3.47

D: P‐selectin, activated integrin *α*_IIb_ *β*3, caspase‐3‐positive cells and ceramide abundance in G*α*_i2_‐deficient female and G*α*_i2_‐deficient male mice				
Parameter	G*α*_i2_‐deficient (female)		G*α*_i2_‐deficient (male)	
	Resting	CoRP	Resting	CoRP
P‐selectin [MFI]	5.28 ± 0.34	22.98 ± 4.65	5.04 ± 0.23	23.34 ± 7.23
Activated integrin *α*_IIb_ *β* [MFI]	5.30 ± 0.13	59.79 ± 16.38	5.44 ± 0.44	44.88 ± 13.68
Caspase‐3‐positive cells [%]	4.10 ± 0.13	8.75 ± 1.52	3.95 ± 0.34	9.43 ± 0.80
Ceramide abundance [MFI]	19.25 ± 1.44	20.13 ± 2.33	20.54 ± 2.43	20.13 ± 2.02

Annexin‐V‐binding, forward scatter, P‐selectin abundance, activated integrin *α*_IIb_ *β*3, caspase‐3‐positive cells, and ceramide abundance in female and male WT and G*α*_i2_‐deficient mice after thrombin and/or CoRP stimulation.

**Figure 1:**
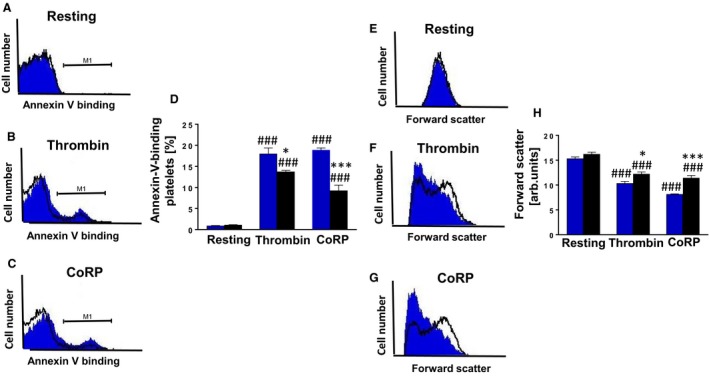
Gα_i2_ participates in the regulation of platelet cell membrane scrambling and platelet volume. *(*A–C) Original histogram overlays of the annexin‐V binding in platelets isolated from WT mice (blue shadow) and G*α*_i2_‐deficient mice (black line) without (A) and with (B) a 10 min treatment with thrombin (0.01 U/mL) or (C) a 10 min treatment with collagen‐related peptide CoRP (5 *μ*g/mL). (D) Arithmetic means ± SD (*n* = 4) of the annexin‐V binding in platelets isolated from WT mice (blue bar) and G*α*_i2_‐deficient mice (black bar) prior to (control) and following a 10 min treatment with thrombin (0.01 U/mL) or a 10 min treatment with collagen‐related peptide CoRP (5 *μ*g/mL). ### (*P *<* *0.001) indicates statistically significant difference from absence of thrombin and CoRP, * (*P *<* *0.05) and *** (*P *<* *0.001) indicates statistically significant difference from WT mice. (E–G) Original histogram overlays of the forward scatter of platelets isolated from WT mice (blue shadow) and G*α*_i2_‐deficient mice (black line) without (E) and with (F) a 10 min treatment with thrombin (0.01 U/mL) or (G) a 10 min treatment with collagen‐related peptide CoRP (5 *μ*g/mL). (H) Arithmetic means ± SD (*n* = 4) of the forward scatter of platelets isolated from WT mice (blue bar) and G*α*_i2_‐deficient mice (black bar) prior to (control) and following a 10 min treatment with thrombin (0.01 U/mL) or a 10 min treatment with collagen‐related peptide CoRP (5 *μ*g/mL). ### (*P *<* *0.001) indicates statistically significant difference from absence of thrombin and CoRP, * (*P *<* *0.05) and *** (*P *<* *0.001) indicates statistically significant difference from WT mice.

Phosphatidylserine exposure, a hallmark of platelet apoptosis, was quantified by flow cytometry analysis using annexin‐V‐binding as described previously (Cao et al. 2017). As illustrated in Figure 1A and D, the percentage of PS‐positive platelets in untreated resting *Gα*_*i2*_^*−/−*^ and WT platelets was not significantly different. Treatment with thrombin and CoRP significantly enhanced the percentage of PS‐positive platelets, an effect significantly less pronounced in *Gα*_*i2*_^*−/−*^ platelets as compared to WT platelets (Fig. 1B–D). Next, we explored whether G*α*_i2_‐mediated platelet apoptosis accompanies cell volume alterations (Cao et al. 2017). As depicted in Figure 1E and H, forward scatter, reflecting platelet volume, was not significantly different in resting platelets of either genotype but was significantly reduced following thrombin and CoRP treatment, an effect significantly less pronounced in *Gα*_*i2*_^*−/−*^ platelets than in WT platelets (Fig. 1F–H).

We then sought to elucidate the mechanism(s) regulating G*α*_i2_‐mediated apoptosis. To this end, we analyzed intracellular Ca^2+^ levels [Ca^2+^]_i_ in murine platelets of either genotype using Fluo3 AM fluorescence as described previously (Cao et al. 2017). As a result, prior to CoRP treatment, [Ca^2+^]_i_ in resting platelets was similar in *Gα*_*i2*_^*−/−*^ platelets (22.73 ± 0.69, a.u.; *n* = 10) and in WT platelets (20.95 ± 0.50, a.u.; *n* = 10). Treatment with CoRP was followed by a profound and significant increase of [Ca^2+^]_i_ in both *Gα*_*i2*_^*−/−*^ (109.3 ± 1.46, a.u., *n* = 10) and WT platelets (105.9 ± 0.69, a.u.; *n* = 10). The increase following CoRP treatment was, however, not significantly different between the two genotypes, thus, ruling out the involvement of Ca^2+^ signaling in G*α*_i2_‐mediated platelet apoptosis.

**Figure 2:**
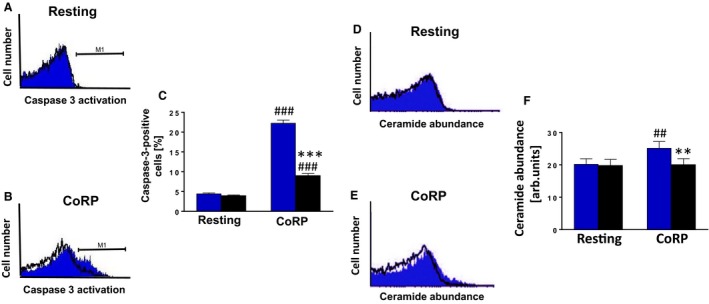
Gα_i2_ participates in the regulation of platelet caspase 3 activation and ceramide formation. (A and B) Original histogram overlays of the caspase 3 activity in platelets isolated from WT mice (blue shadow) and G*α*_i2_‐deficient mice (black line) without (A) and with (B) a 10 min treatment with collagen‐related peptide CoRP (5 *μ*g/mL). (C) Arithmetic means ± SD (*n* = 6) of the caspase 3 activity (arbitrary units) in platelets isolated from WT mice (blue bar) and G*α*_i2_‐deficient mice (black bar) prior to (control) and following a 10 min treatment with collagen‐related peptide CoRP (5 *μ*g/mL). ^###^ (*P *<* *0.001) indicates statistically significant difference from absence of CoRP, *** (*P *<* *0.001) indicates statistically significant difference from WT mice. (D and E) Original histogram overlays of the ceramide abundance of platelets isolated from WT mice (blue shadow) and G*α*_i2_‐deficient mice (black line) without (D) and with (E) a 10 min treatment with collagen‐related peptide CoRP (5 *μ*g/mL). (F) Arithmetic means ± SD (*n* = 4) of the ceramide abundance of platelets isolated from WT mice (blue bar) and G*α*_i2_‐deficient mice (black bar) prior to (control) and following a 10 min treatment with collagen‐related peptide CoRP (5 *μ*g/mL). ^##^ (*P *<* *0.01) indicates statistically significant difference from absence of CoRP, ** (*P *<* *0.01) indicates statistically significant difference from WT mice.

Next, we explored whether other putative mechanisms underpin the regulation of G*α*_i2_‐mediated platelet apoptosis. To this end, caspase 3 fluorescence (Cao et al. 2017) was measured and was similar in resting platelets of either genotype (Fig. 2A and C), but was significantly upregulated after CoRP treatment, an effect which was significantly blunted in *Gα*_*i2*_^*−/−*^ platelets as compared to WT platelets (Fig. 2B and C).

As has previously been shown, hyperosmotic shock triggered a significant increase in platelet annexin‐V‐binding, an effect probably involving ceramide formation (Gatidis et al. 2010). We thus examined whether ceramide formation is altered in G*α*_i2_‐deficient platelets as compared to WT platelets. As illustrated in Figure 2D and F, ceramide abundance was similar in resting platelets. CoRP treatment elicited a significant increase in ceramide abundance in WT platelets as compared to *Gα*_*i2*_^*−/−*^ platelets (Fig. 2E and F), pointing to the involvement of sphingomyelinase activation in the triggering of G*α*_i2_‐mediated platelet apoptosis.

**Figure 3:**
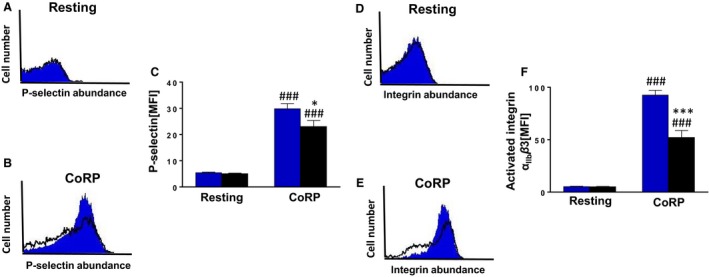
Gα_i2_ participates in the regulation of platelet degranulation and integrin α_II__b_ *β*3 activation. (A and B) Original histogram overlays of P‐selectin‐related fluorescence reflecting degranulation in platelets isolated from WT mice (blue shadow) and G*α*_i2_‐deficient mice (black line) without (A) and with (B) a 15 min treatment with collagen‐related peptide CoRP (2 *μ*g/mL). (C) Arithmetic means ± SD (*n* = 6) of the P‐selectin‐related fluorescence (arbitrary units) in platelets isolated from WT mice (blue bar) and G*α*_i2_‐deficient mice (black bar) prior to (control) and following a 15 min treatment with collagen‐related peptide CoRP (2 *μ*g/mL). ^###^ (*P *<* *0.001) indicates statistically significant difference from absence of CoRP, * (*P *<* *0.05) indicates statistically significant difference from WT mice. (D and E) Original histogram overlays of the *α* _II__b_ *β*3 integrin‐related fluorescence in platelets isolated from WT mice (blue shadow) and G*α*_i2_‐deficient mice (black line) without (D) and with (E) a 15 min treatment with collagen‐related peptide CoRP (2 *μ*g/mL). (F) Arithmetic means ± SD (*n* = 6) of the *α* _II__b_ *β*3 integrin‐related fluorescence (arbitrary units) in platelets isolated from WT mice (blue bar) and G*α*_i2_‐deficient mice (black bar) prior to (control) and following a 15 min treatment with collagen‐related peptide CoRP (2 *μ*g/mL). ^###^ (*P *<* *0.001) indicates statistically significant difference from absence of CoRP, *** (*P *<* *0.001) indicates statistically significant difference from WT mice.

We then tested whether G*α*_i2_ similarly mediates platelet degranulation in response to CoRP. To this end, P‐selectin expression was similar in untreated resting and *Gα*_*i2*_^*−/−*^ platelets (Fig. 3A and C). Exposure to CoRP significantly increased P‐selectin expression (Cao et al. 2017) on the platelet surface reflecting enhanced degranulation; this effect was significantly reduced in *Gα*_*i2*_^*−/−*^ platelets as compared to WT platelets (Fig. 3B and C). Next, we explored whether active integrin *α*_IIb_ *β*3 is different in the two genotypes. As a result, active integrin *α*_IIb_ *β*3 was similar in WT and *Gα*_*i2*_^*−/−*^ platelets (Fig. 3D and F). In addition, CoRP treatment further stimulated active integrin *α*_IIb_ *β*3 (Cao et al. 2017) at the platelet surface, an effect that was again significantly attenuated in *Gα*_*i2*_^*−/−*^ platelets (Fig. 3E and F). Thus, G*α*_i2_ is dichotomously involved in both platelet degranulation and apoptosis following agonist exposure.

## Discussion

Previous studies have shown that the effect of CoRP on degranulation, PS exposure, cell volume, and *α*_IIb_ *β*3 integrin abundance is mediated, at least in part, by increased [Ca^2+^]_i_ (Varga‐Szabo et al. 2009; Lang et al. 2013), which is known to trigger platelet activation and stimulate arterial thrombosis (Lang et al. 2013). Surprisingly, elevation of [Ca^2+^]_i_ was similar in platelets of either genotype suggesting that G*α*_i2_ modifies platelet activation and apoptosis by interfering with mechanisms other than Ca^2+^ entry.

Acid sphingomyelinase degrades membrane sphingomyelin and generates ceramide, which is responsible for cell membrane scrambling and degranulation in a variety of cell types; this lipid signaling is vital in the pathophysiology of several cardiovascular diseases (Lang et al. 2015). Remarkably, both genetic deficiency and pharmacological inhibition of acid sphingomyelinase has been shown to thwart platelet secretion and PS exposure independently of Ca^2+^ signaling, purportedly due to a signaling defect downstream of phospholipase C activation (Munzer et al. 2014). Ceramide metabolism further involves ceramidases which has been reported to ameliorate CoRP‐triggered glycoprotein VI‐dependent platelet aggregation and thrombus formation (Munzer et al. 2018). Along these lines, it is tempting to speculate that PS‐exposure and degranulation of platelets mediated by G*α*_i2_‐sensitive ceramide generation is a possible mechanism linking previous observations of tissue injury and thrombosis after experimental ischemia‐reperfusion.

In conclusion, the present study sheds light on a novel function of G*α*_i2_ protein, that is, the regulation of platelet apoptosis, a process, at least in part, mediated by agonist‐sensitive ceramide formation. Our observations on G*α*_i2_‐mediated platelet survival may have pathophysiological implications in thrombo‐inflammatory conditions. Further investigations are warranted to dissect the relevance of this mechanism in platelet‐related disorders in humans.

## Conflict of Interest

The authors state that they have no conflict of interest.

## References

[phy213841-bib-0001] Bissinger, R. , E. Lang , M. Ghashghaeinia , Y. Singh , C. Zelenak , B. Fehrenbacher , et al. 2016 Blunted apoptosis of erythrocytes in mice deficient in the heterotrimeric G‐protein subunit Galphai2. Sci. Rep. 6:30925. 27499046 10.1038/srep30925 PMC4976336

[phy213841-bib-0002] Cao, H. , A. A. M. Bhuyan , A. T. Umbach , R. Bissinger , M. Gawaz , and F. Lang . 2017 Inhibitory effect of afatinib on platelet activation and apoptosis. Cell. Physiol. Biochem. 43:2264–2276. 29073606 10.1159/000484377

[phy213841-bib-0003] Devanathan, V. , I. Hagedorn , D. Kohler , K. Pexa , D. Cherpokova , P. Kraft , et al. 2015 Platelet Gi protein Galphai2 is an essential mediator of thrombo‐inflammatory organ damage in mice. Proc. Natl. Acad. Sci. USA 112:6491–6496. 25944935 10.1073/pnas.1505887112 PMC4443332

[phy213841-bib-0004] Gachet, C. 2012 P2Y(12) receptors in platelets and other hematopoietic and non‐hematopoietic cells. Purinergic Signal. 8:609–619. 22528678 10.1007/s11302-012-9303-x PMC3360102

[phy213841-bib-0005] Gatidis, S. , O. Borst , M. Foller , and F. Lang . 2010 Effect of osmotic shock and urea on phosphatidylserine scrambling in thrombocyte cell membranes. Am. J. Physiol. Cell Physiol. 299:C111–C118. 20237147 10.1152/ajpcell.00477.2009

[phy213841-bib-0006] Hilger, D. , M. Masureel , and B. K. Kobilka . 2018 Structure and dynamics of GPCR signaling complexes. Nat. Struct. Mol. Biol. 25:4–12. 29323277 10.1038/s41594-017-0011-7 PMC6535338

[phy213841-bib-0007] Lang, F. , P. Munzer , M. Gawaz , and O. Borst . 2013 Regulation of STIM1/Orai1‐dependent Ca2+ signalling in platelets. Thromb. Haemost. 110:925–930. 23846758 10.1160/TH13-02-0176

[phy213841-bib-0008] Lang, E. , R. Bissinger , E. Gulbins , and F. Lang . 2015 Ceramide in the regulation of eryptosis, the suicidal erythrocyte death. Apoptosis 20:758–767. 25637185 10.1007/s10495-015-1094-4

[phy213841-bib-0009] Lebois, M. , and E. C. Josefsson . 2016 Regulation of platelet lifespan by apoptosis. Platelets 27:497–504. 27100842 10.3109/09537104.2016.1161739

[phy213841-bib-0010] Liu, G. , G. Liu , H. Chen , O. Borst , M. Gawaz , A. Vortkamp , et al. 2015 Involvement of Ca2+ activated Cl‐ channel Ano6 in platelet activation and apoptosis. Cell. Physiol. Biochem. 37:1934–1944. 26584292 10.1159/000438554

[phy213841-bib-0011] Liu, G. , G. Liu , M. Chatterjee , A. T. Umbach , H. Chen , M. Gawaz , et al. 2016 Influence of gamma‐secretase inhibitor 24‐Diamino‐5‐Phenylthiazole DAPT on platelet activation. Cell. Physiol. Biochem. 38:726–736. 26871421 10.1159/000443029

[phy213841-bib-0012] Lopez‐Aranda, M. F. , I. Navarro‐Lobato , J. F. Lopez‐Tellez , E. Blanco , M. Masmudi‐Martin , and Z. U. Khan . 2008 Activation of caspase‐3 pathway by expression of sGalphai2 protein in BHK cells. Neurosci. Lett. 439:37–41. 18502580 10.1016/j.neulet.2008.04.078

[phy213841-bib-0013] Munzer, P. , O. Borst , B. Walker , E. Schmid , M. A. Feijge , J. M. Cosemans , et al. 2014 Acid sphingomyelinase regulates platelet cell membrane scrambling, secretion, and thrombus formation. Arterioscler. Thromb. Vasc. Biol. 34:61–71. 24233488 10.1161/ATVBAHA.112.300210

[phy213841-bib-0014] Munzer, P. , S. Mittelstadt , S. Geue , M. C. Manke , B. Walker‐Allgaier , F. Lang , et al. 2018 Ceramidase critically affects GPVI‐dependent platelet activation and thrombus formation. Biochem. Biophys. Res. Commun. 496:792–798. 29395079 10.1016/j.bbrc.2018.01.155

[phy213841-bib-0015] Offermanns, S. 2006 Activation of platelet function through G protein‐coupled receptors. Circ. Res. 99:1293–1304. 17158345 10.1161/01.RES.0000251742.71301.16

[phy213841-bib-0016] Quach, M. E. , W. Chen , and R. Li . 2018 Mechanisms of platelet clearance and translation to improve platelet storage. Blood 131:1512–1521. 29475962 10.1182/blood-2017-08-743229 PMC5887765

[phy213841-bib-0017] Rudolph, U. , M. J. Finegold , S. S. Rich , G. R. Harriman , Y. Srinivasan , P. Brabet , et al. 1995 Ulcerative colitis and adenocarcinoma of the colon in G alpha i2‐deficient mice. Nat. Genet. 10:143–150. 7663509 10.1038/ng0695-143

[phy213841-bib-0018] Squires, K. E. , C. Montanez‐Miranda , R. R. Pandya , M. P. Torres , and J. R. Hepler . 2018 Genetic analysis of rare human variants of regulators of G protein signaling proteins and their role in human physiology and disease. Pharmacol. Rev. 70:446–474. 29871944 10.1124/pr.117.015354 PMC5989036

[phy213841-bib-0019] Towhid, S. T. , E. M. Schmidt , E. Schmid , P. Munzer , S. M. Qadri , O. Borst , et al. 2011 Thymoquinone‐induced platelet apoptosis. J. Cell. Biochem. 112:3112–3121. 21688304 10.1002/jcb.23237

[phy213841-bib-0020] Varga‐Szabo, D. , A. Braun , and B. Nieswandt . 2009 Calcium signaling in platelets. J. Thromb. Haemost. 7:1057–1066. 19422456 10.1111/j.1538-7836.2009.03455.x

[phy213841-bib-0021] Wiege, K. , S. R. Ali , B. Gewecke , A. Novakovic , F. M. Konrad , K. Pexa , et al. 2013 Galphai2 is the essential Galphai protein in immune complex‐induced lung disease. J. Immunol. 190:324–333. 23225882 10.4049/jimmunol.1201398

